# Oral History. Interviews mit Psychiatriepatient*innen und Bewohner*innen von Einrichtungen der Behindertenhilfe – ein Praxisbericht

**DOI:** 10.1007/s00048-024-00378-1

**Published:** 2024-02-27

**Authors:** Frank Sparing, Nils Löffelbein, Uta Hinz

**Affiliations:** 1 Institut für Geschichte, Theorie und Ethik der Medizin, Centre for Health and Society, Heinrich-Heine-Universität Düsseldorf, Moorenstr. 5, 40225 Düsseldorf, Deutschland; 2 Institut für Neuere Geschichte, Heinrich-Heine-Universität Düsseldorf, Universitätsstraße 1, 40225 Düsseldorf, Deutschland

## Warum Oral History mit Patient*innen psychiatrischer Kliniken und Bewohner*innen von Einrichtungen der Behindertenhilfe?

Seit dem Beginn der intensiven Medienberichterstattung über die gewaltgeprägten Umstände der Heim- und Anstaltsunterbringung von Kindern und Jugendlichen in der frühen Bundesrepublik Anfang der 2000er Jahre wurde eine Vielzahl medizinhistorischer Untersuchungen zur Thematik angestoßen. Diese widmeten sich erstmals intensiver den Lebensverhältnissen in psychiatrischen Anstalten und Einrichtungen der Behindertenhilfe in der Zeit von 1945 bis 1975 (Kersting & Schmuhl 2018; Siebert et al. 2016; Schäfer-Walkmann & Hein 2014), wobei hier vor allem die Leid- und Unrechtserfahrungen der damals minderjährigen Patient*innen und Bewohner*innen in den Fokus gerückt sind (Fehlemann & Sparing 2017; Sparing 2018).

In neueren Studien wurde dabei erstmals verstärkt auf Zeitzeug*innen-Interviews als Quelle zurückgegriffen (vgl. etwa Winkler & Schmuhl 2013; Siebert et al. 2016; Silberzahn-Jandt 2019), womit auch in Psychiatriegeschichte und Heimkinderforschung die Betroffenenperspektive sichtlich an Bedeutung gewonnen hat. Methoden der Oral History werden in diesem Feld erst seit einigen Jahren für die Forschungspraxis fruchtbar gemacht. So galt es noch Anfang der 1980er Jahre wissenschaftlich als wenig sinnvoll, Menschen mit geistigen Beeinträchtigungen im Rahmen von Interviews zu befragen, da „geistig Behinderte“ auch in der Forschung als „Prototyp des Nicht-Befragbaren“ wahrgenommen wurden (Laga 1982: 228). Mittlerweile werden jedoch auch geistig beeinträchtigte Menschen als wichtige Zeug*innen anerkannt, deren Erfahrungen und Agency es zu berücksichtigen gilt (George 2013: 79f.; George 2008).

In der neueren medizinhistorischen Forschung zur Heim- und Anstaltsunterbringung Minderjähriger bilden Interviews mit ehemaligen Patient*innen und Bewohner*innen sogar vielfach die einzige Quelle zum Einrichtungsalltag (Beyer et al. 2022; Fangerau et al. 2021), geben doch die überlieferten Patient*innen‑/Bewohner*innenakten zumeist ausschließlich den Blick der „Institution“ wieder – also denjenigen von Behörden, Ärzt*innen und Pflegenden. Der Erfahrungs- und Wahrnehmungshorizont der betreuten Menschen bleibt hingegen ebenso ausgeblendet wie die Wirkung bürokratischer, pflegerischer und medizinischer Routinen oder von eventuellem Fehlverhalten des Fachpersonals.

Trotz der mittlerweile zahlreich vorliegenden Einrichtungsstudien hat eine theoretische Verortung und wissenschaftliche Diskussion über die spezifischen Herausforderungen der Oral History mit vulnerablen Gruppen in der deutschsprachigen Heim- und Psychiatriegeschichtsforschung bislang kaum stattgefunden. Im angloamerikanischen Raum existieren dagegen bereits seit einigen Jahrzehnten anleitende Forschungsarbeiten, die sich dezidiert mit den Umständen, Möglichkeiten, aber auch methodologischen Grenzen von Oral-History-Interviews mit beeinträchtigten Menschen auseinandersetzen (Atkinson 2000; Hirsch 1995). In Deutschland ist eine diesbezügliche Systematisierung des eigenen Forschungssettings und -designs und der verwendeten Methoden bislang hingegen kaum erfolgt. So sind etwa die spezifischen Anforderungen und Bedürfnisse im Umgang mit unterschiedlichen Formen und Graden von Beeinträchtigungen – also körperlichen und geistigen Behinderungen, Lernbehinderungen, Sinnesstörungen sowie Schwerstmehrfachbehinderungen – in vorliegenden Forschungsprojekten bislang wenig differenziert worden. Auch eine Auseinandersetzung mit dem Paradigma der Inklusiven Forschung, also dem gemeinsamen Forschen von Wissenschaftler*innen und Interviewpartner*innen, hat in Deutschland erst begonnen (Kremser 2017: 138). Dabei zeigen sich gerade in der Gesprächssituation mit beeinträchtigten Menschen besondere Anforderungen und Erfahrungen, deren Reflexion auch für die methodische Diskussion von Oral History in weiteren (medizin-)historischen Kontexten fruchtbar sein dürfte.

Der folgende Beitrag zielt nicht darauf ab, eine umfassende theoretische Verortung der Oral-History-Methode in der Psychiatrie- und Heimkinderforschung vorzunehmen. Vielmehr versteht er sich als Praxisbericht, in dessen Zentrum besondere Gesprächskonstellationen in Zeitzeug*inneninterviews stehen, die im Rahmen von Forschungsprojekten des Instituts für Geschichte, Theorie und Ethik der Medizin (IGTEM) Düsseldorf durchgeführt wurden. Nachfolgend sollen hier daher lediglich explorativ einige methodische und praktische Herausforderungen skizziert werden, die für die Interaktionen mit Menschen mit geistigen Beeinträchtigungen in der Oral-History-Forschung als grundlegend erscheinen. Im Projekt „Aufarbeitung und Dokumentation der Geschichte der Menschen mit Behinderungen und psychiatrischen Erkrankungen in Einrichtungen des Landschaftsverbandes Rheinland seit 1945“ (Sparing 2018) wurden acht Interviews mit ehemaligen Patient*innen psychiatrischer Anstalten im Rheinland umgesetzt, die vor 1970 dort untergebracht waren. Im Rahmen des 2021 abgeschlossenen Projektverbunds „Wissenschaftliche Aufarbeitung von Leid und Unrecht, das Kinder und Jugendliche in Einrichtungen der Psychiatrie und Behindertenhilfe in BRD (1945–1975) und DDR (1945–1989) erlitten haben“ (Fangerau et al. 2021) konnten in drei der untersuchten westdeutschen Einrichtungen der Behindertenhilfe insgesamt 18 Interviews geführt werden. Der Grad der geistigen Beeinträchtigung war bei den Interviewpartner*innen in den hier vorgestellten Projekten dabei sehr unterschiedlich ausgeprägt. So ließ sich bei einigen Personen der eigentliche Grund für ihre Einweisung in eine „Anstalt“ rückblickend nicht mehr rekonstruieren. Im Untersuchungszeitraum waren sie zumeist aufgrund sozial devianter Verhaltensweisen in Heime oder Psychiatrien eingewiesen worden, die bis weit in die 1970er Jahre üblicherweise mit der unscharfen Sammeldiagnose „Schwachsinn“ belegt wurden. Als Kinder und Jugendliche verbrachten diese Personen jedoch mehrheitlich nur einige Jahre in den Einrichtungen. Andere Interviewpartner*innen sind aufgrund einer starken geistigen Beeinträchtigung hingegen bis in die Gegenwart auf engmaschige Hilfen angewiesen und leben noch immer in der stationären Einrichtung ihrer Kindheit oder Jugend. Insgesamt wurden in den vorliegenden Forschungssettings ausschließlich Gespräche mit Erwachsenen geführt, deren Kindheit oder Jugend in Heimen beziehungsweise Psychiatrien bereits mehrere Jahrzehnte zurücklag.

## Methodische und praktische Herausforderungen

Oral-History-Projekte haben meist sehr verschiedene Ausgangs- und Rahmenbedingungen, sodass die methodischen Zugänge je nach Anlage des Projekts variieren können. Erkenntnisinteresse, Fragestellung, Befragungsumstände und Interviewpartner*innen sind oftmals derart heterogen, dass ein starres Methodenkonzept nicht sinnvoll erscheint (Stöckle 1990: 132) und die Vorgehensweise in der praktischen Durchführung und theoretischen Reflexion stets aufs Neue erarbeitet werden muss (Brüggemeier 1984: 199).

Bei der Durchführung von narrativen Interviews mit kognitiv beeinträchtigten Menschen in stationären Langzeiteinrichtungen sind zudem Rahmenbedingungen und Gesprächskonstellationen anzutreffen, die bereits im Vorfeld spezielle Fragen aufwerfen – zum Beispiel, wie eine „informierte Zustimmung“ der Zeitzeug*innen praktisch sicherzustellen ist (Siebert et al. 2016: 61). Mit Blick auf medizinische Aspekte sind mögliche Beeinträchtigungen – etwa durch Langzeitfolgen verabreichter Medikamente – ebenso zu berücksichtigen wie individuelle Besonderheiten von Kognition und Kommunikation (Schäfer-Walkmann & Hein 2014: 20–22). In der Gesprächssituation resultieren daraus nicht allein methodische Herausforderungen einer möglichen kommunikativen Anpassung an individuelle Bedürfnisse – etwa in Form von Leichter Sprache (Siebert et al. 2016: 17). Sehr eng sind diese auch mit ethischen Fragen wie der Vermeidung kommunikativer Hierarchien und möglicher Retraumatisierungen verbunden.^1^ Wichtig erschien in den durchgeführten Projekten daher, zusätzlich zu einer kurzen schriftlichen Information im Umfeld der Einwilligungserklärung den Interviewpartner*innen eingangs eine Orientierung über Hintergründe und Ziele des Gesprächs zu geben, um Vertrauen aufzubauen. Die Interviews wurden aus diesem Grund stets mit einer kurzen Projektvorstellung begonnen, um Gelegenheit für Nachfragen und Diskussion zu geben.

Methodisch bietet es sich an, offene, „narrative Interviews“ (Schütze 1976: 159–260) zu führen, in denen die Gesprächspartner*innen die thematischen Schwerpunkte ihrer Erzählung selbstständig gewichten können (Helfferich 2011; Lucius-Hoene & Deppermann 2004). Gerade bei der historischen Auseinandersetzung mit Missbrauchs- und Gewalterfahrungen stehen zudem die persönliche „Deutung, Einordnung und Bewertung der Erfahrungen“ im Zentrum des Interesses (Kersting & Schmuhl 2018: 19). Die Erzählung sollte daher von dem/der Interviewenden weder unterbrochen noch durch Nachfragen angeregt werden.

Bei Interviews mit geistig beeinträchtigten Menschen muss mit einer eingeschränkten Kommunikationsfähigkeit der Gesprächspartner*innen gerechnet werden. Teilweise ist ein Interview sogar nur mithilfe von Betreuenden möglich, wodurch Verlauf und Ergebnis zusätzlich beeinflusst werden können. Medizinische Einflussfaktoren gelten in zeitzeugenbasierten Studien bislang vor allem als praktische, kommunikative Herausforderung. Seltener wird dagegen methodisch diskutiert, inwiefern sie bei bestimmten Gesprächspartner*innen als wahrnehmungs-, erinnerungs- und kommunikationsrelevante Faktoren wirken und in eine Auswertung einbezogen werden können (Schäfer-Walkmann & Hein 2014: 21–22).

Eine besondere Rahmenbedingung ist zudem durch die soziokulturelle Umfeldkonstellation der Interviewpartner*innen gegeben. In der Regel lebten und leben speziell die befragten Heimbewohner*innen jahrzehntelang in derselben stationären Einrichtung, mit der ihre Sozialisation und Lebenserfahrung eng verzahnt ist. Zugespitzt formuliert: Es besteht ein teils lebenslanges Prägungs- und Abhängigkeitsverhältnis, das durch eine bis in die 1980er Jahre institutionell stark abgeschlossene Lebenswelt strukturiert und in den zahlreichen konfessionell geführten Einrichtungen in der Regel religiös überformt ist. Die Spezifika der Unterbringung nehmen auch deutlichen Einfluss auf Verlauf und Ergebnis der Interviews mit ehemaligen Patient*innen/Bewohner*innen. So ist eine zeitliche Einordnung von Erlebnissen und Ereignissen oft problematisch, da sich durch sich wiederholende Tagesabläufe mit jahrzehntelang immer gleichen Routinen in einer geschlossenen Einrichtung (Goffman 1973) Erinnerungen an strukturierende Einschnitte kaum herausbilden können (Sparing 2020; Löffelbein 2021).

Die Prägung von Zeitzeug*innen durch eine lebenslange und enge Verbindung zu einer Institution kann sich jedoch unterschiedlich auswirken. Während oftmals eine extreme Durchdringung des biografischen Narrativs und der individuellen Sinndeutung durch die institutionelle Logik der „Anstalt“ feststellbar ist, entwickelten Patient*innen/Bewohner*innen in anderen Fällen durchaus einen „rebellischen“ beziehungsweise „widerständigen“ Habitus (Goffman 1973: 64–70; Siebert et al. 2016: 126). Es zeigt sich zudem, dass sich die je nach Gesprächspartner*in unterschiedlich stark ausgestaltete individuelle Sinnkonstruktion auch auf die Interviewauswertung auswirkt. Während manche Befragte detailliertere Erzählstränge entwickelten, blieb der Modus des Erzählens bei anderen eher reaktiv und knapp. Selbst in Interviews mit sprachlich und kognitiv eingeschränkten Gesprächspartner*innen kann sich aber eine emotional-körperliche Kommunikationsebene entwickeln, welche die Deutung und Einordnung auch sehr kurzer verbaler Aussagen zulässt. Erforderlich ist dabei, nicht allein auf schriftliche Interviewtranskriptionen zurückzugreifen, sondern auch auf Audio- oder Videoaufzeichnungen.

## Weiterführende Fragen

Mit Blick auf eine theoretisch geleitete Methodenreflexion von Zeitzeug*inneninterviews lassen sich aus den vorliegenden Erfahrungen einige weiterführende Fragen ableiten. Als eine besondere Herausforderung erweist sich zunächst die zentrale Frage, wie eine zeitzeugengestützte Aufklärung von erlebter Gewalt erfolgen kann, ohne die Befragten zu belasten oder sogar zu re-traumatisieren (Abrams 2016: 92f.; Rosenthal 1995: 172).

Einen weiteren Punkt bildet die Frage, durch welche methodischen Vorgehensweisen die Kommunikation mit Zeitzeug*innen lebensweltgerecht angepasst werden kann. Grenzen zeigen sich etwa, wenn bei Menschen ein Erzählen nicht gewohnten Formen und Abläufen folgt. Wie skizziert, erfordert die Anpassung an individuelle Kommunikationsformen und -bedürfnisse nicht nur eine hohe Sensibilität im Verlauf des Interviews, sondern auch eine differenzierte Analyse verbaler wie nonverbaler Kommunikation im Auswertungsprozess.

Ein letzter Punkt bezieht sich schließlich auf den Umgang mit spezifischen Rahmenbedingungen und Einflussfaktoren, die in sehr verschiedenen medizinhistorischen Kontexten eine Rolle spielen können und die direkt mit der grundsätzlichen Frage nach Formen und Funktionsweisen individueller Erinnerung verbunden sind. Wie hier gezeigt, können neben der „anstaltsförmigen“ Langzeitunterbringung auch Langzeitwirkungen von Medikamenten die Folgen angewandter „Therapien“ oder Hospitalisierungsschäden die Wahrnehmung und Erinnerung beeinflussen – ebenso individuelle (z. B. kognitive) Beeinträchtigungen oder die Symptomatik bestimmter psychiatrischer Erkrankungen.

## Kurzausblick

Oral-History-Interviews stellen mittlerweile einen wichtigen Zugang zur historischen Aufarbeitung von Anstalts- und Heimerfahrungen dar. Ein zentrales Ergebnis der skizzierten Projekte ist die starke Präsenz vor allem physischer Gewalt in den Zeitzeug*inneninterviews, während gewalttätiges Verhalten in den Schriftquellen der Einrichtungen meist unerwähnt bleibt (vgl. z. B. Schäfer-Walkmann & Hein 2014: 138–149; Siebert et al. 2016: 116–136; Silberzahn-Jandt 2019: 98–106) – ein Befund, der die hohe Relevanz der Oral History für eine Alltags- und Erfahrungsgeschichte von Psychiatrie und Behindertenhilfe nach 1945 unterstreicht. Wie skizziert, sind in diesem Zusammenhang nonverbale oder nicht in ein Narrativ eingeordnete Aussagen von Menschen mit psychischen Erkrankungen und geistigen Behinderungen methodisch durchaus in die Auswertung integrierbar und für eine umfassendere Bewertung sogar von zentraler Bedeutung. Entsprechende Methodik aus den bisherigen Praxiserfahrungen heraus weiterzuentwickeln wäre nicht allein für die zukünftige Forschung im Bereich Heim- oder Psychiatriegeschichte sinnvoll. Zu diskutieren wäre auch ihre Übertragbarkeit auf weitere erfahrungsorientierte (medizin-)historische Untersuchungsfelder.

## Footnotes

1 Etwa indem das Auslassen sensibler, belastender Themen durch Interviewte akzeptiert wird, ein Interview unterbrochen oder auch abgebrochen werden kann sowie dadurch, dass insbesondere psychosoziale Hilfe bereitgestellt wird (Siebert et al. 2016: 58f.).
